# Comparing clinical outcomes of dogs suffering from degenerative lumbosacral stenosis upon surgical or nonsurgical treatment

**DOI:** 10.18849/ve.v8i2.575

**Published:** 2023-06-08

**Authors:** Kristy Goh+

**Keywords:** DOGS, DEGENERATIVE LUMBOSACRAL STENOSIS, DORSAL LAMINECTOMY, EPIDURAL STEROID INJECTION, CAUDA EQUINA, DISC DEGENERATION, TRANSARTICULAR FUSION, TRANSARTICULAR STABILISATION, CANINE DLSS, FORAMINOTOMY

## Abstract

**PICO question:**

In dogs suffering from degenerative lumbosacral stenosis (DLSS), is surgical treatment more effective than nonsurgical therapy in reducing lumbosacral pain and neurological dysfunction in the long-term?

**Clinical bottom line:**

**Category of research:**

Treatment.

**Number and type of study designs reviewed:**

Two papers were critically reviewed. They were prospective and retrospective studies.

**Strength of evidence:**

Weak.

**Outcomes reported:**

Besides the two studies, there are no other studies currently available that directly compare long-term clinical outcome of patients that have undergone nonsurgical and surgical treatment respectively.

In the study comparing clinical outcome of nonsurgical treatment by epidural steroid injection (ESI) and surgical treatment of degenerative lumbosacral stenosis, dogs were classified into clinical severity groups ranging from mild to moderate to severe. Mild cases demonstrated degenerative lumbosacral stenosis (DLSS) compatible clinical signs such as lumbosacral pain, reluctance to climb stairs / jump / raise up, lameness and muscle atrophy but no neurological deficits. Moderate cases presented DLSS compatible clinical signs in combination with neurological deficits such as reduced flexor withdrawal, proprioceptive deficits and nerve root signature. Severe cases demonstrated DLSS compatible clinical signs with more severe neurological deficits such as tail paresis and absent perineal reflex. Clinical outcomes were considered complete if clinical signs had resolved at follow-up consultations, partial if there was substantial but incomplete improvement in clinical signs and failed if the dog did not improve or deteriorated further. Improvements in patient condition were measured in terms of clinical outcome grading which is in relation to the initial clinical severity group assigned to each dog. Improvement after single dose of ESI was seen in 27/32 dogs, with 17/22 (after accounting for four dogs whose owners have refused further treatment, five dogs lost to follow-up after re-check as well as one dog whose owners have opted for repeated ESI instillations) relapsing within 6 months. All 17 of these dogs that suffered a relapse after single ESI subsequently underwent surgical treatment and demonstrated improvement in clinical signs, with a complete response seen in eight dogs and a partial response seen in nine dogs.

In the study comparing clinical outcome of conservative treatment of exercise restriction with phenylbutazone administration and surgical treatment of degenerative lumbosacral stenosis, outcomes were classified as good in dogs that regained preoperative activity levels; acceptable in dogs with persistent abnormality or requiring continued medication though otherwise active, and poor in all other cases. Out of 16 dogs treated surgically, 11 were treated by dorsal lumbosacral laminectomy and excision of the dorsal portion of the lumbosacral disc, while the other five had additional unilateral facetectomy to decompress the seventh lumbar nerve. Out of the 11 dogs treated with dorsal lumbosacral laminectomy and excision of the dorsal portion of the lumbosacral disc, 6/11 (54.5%) of dogs were deemed to have a good outcome, while 3/11 (27.3%) of dogs were deemed to have an acceptable outcome. Out of the five dogs treated with dorsal lumbosacral laminectomy and excision of the dorsal portion of the lumbosacral disc with additional unilateral facetectomy, 3/5 (60%) of dogs were deemed to have an acceptable outcome. The outcome of conservative treatment was deemed good in 8/16 (50%) of dogs in the conservative treatment group.

**Conclusion:**

There is evidence suggesting that both nonsurgical and surgical treatments can improve clinical outcomes and reduce lower back pain and neurological deficits. However, based on the current limited literature, it cannot be ascertained whether surgical treatments are more effective than nonsurgical treatments in improving long-term clinical outcomes and vice versa. In the study that tested the efficacy of epidural steroid injection, only a single dose of steroids was given in this study, making it a potential reason for the high rate of relapse following nonsurgical treatment. For surgical treatment of DLSS, the type of surgical procedure chosen would also depend on the part of the lumbosacral region which fails and leads to compression. In conclusion, randomised controlled trials that compare different forms of nonsurgical treatment with surgical treatment for dogs with DLSS caused by different underlying factors need to be conducted to properly address the PICO question.

## How to apply this evidence in practice

The application of evidence into practice should take into account multiple factors, not limited to: individual clinical expertise, patient’s circumstances and owners’ values, country, location or clinic where you work, the individual case in front of you, the availability of therapies and resources.

Knowledge Summaries are a resource to help reinforce or inform decision making. They do not override the responsibility or judgement of the practitioner to do what is best for the animal in their care.

## The evidence

Only two studies compared the outcome after nonsurgical and surgical treatment and was thus included in this Knowledge Summary.

Several studies have elaborated on the efficacy of surgical procedures such as dorsal laminectomies, however, a lack of controlled studies prohibits direct comparison between those that receive nonsurgical treatment and surgical treatment. Among the studies which have surfaced through the literature search but do not address the PICO question, were those which evaluated the success of solely surgical treatment of degenerative lumbosacral stenosis (DLSS). Most papers focused on cauda equina syndrome only or were review papers on DLSS pathogenesis, diagnosis, and management, with little focus on the treatment aspect of DLSS.

The studies which directly answered the PICO question were prospective and retrospective studies, which rank low in the evidence hierarchy.

## Summary of the evidence

**Gomes et al. (2020):** 

Population:	Dogs presented to the neurology service at a single referral hospital between February 2017 and May 2019, with clinical signs compatible with degenerative lumbosacral stenosis (DLSS) were recruited. Inclusion criteria: Clinical confirmation of DLSS through compatible clinical signs. Magnetic resonance imaging (MRI) evidence of intervertebral foraminal stenosis with identification of L7 nerve root enlargement and / or lumbosacral vertebral canal stenosi. Exclusion criteria: Dogs presenting with concomitant relevant orthopaedic, neoplastic, inflammatory, developmental conditions or evidence of L7-S1 intervertebral disc extrusion. Dogs that were recruited were classified into clinical severity groups. Mild cases demonstrated DLSS compatible clinical signs such as lumbosacral pain, reluctance to climb stairs / jump / raise up, lameness and muscle atrophy but no neurological deficits. Moderate cases presented DLSS compatible clinical signs in combination with neurological deficits such as reduced flexor withdrawal, proprioceptive deficits and nerve root signature. Severe cases demonstrated DLSS compatible clinical signs with more severe neurological deficits such as tail paresis and absent perineal reflex.
Sample size:	41 dogs.
Intervention details:	41 dogs underwent an epidural steroid injection (ESI) of methylprednisolone acetate (Depo-Medrone 40 mg/mL, Pfizer) into the lumbosacral epidural space, following a protocol of 1 mg/kg with a minimal volume of 0.5 mL. Nine dogs were lost to follow-up. Surgical decompression was performed in 17 out of the remaining 32 dogs following a minimum period of 2 weeks following ESI, when unsuccessful or after relapse of clinical signs.
Study design:	Prospective study.
Outcome studied:	Clinical outcome as assessed by a board-certified neurologist on follow-up consultations. Outcomes were based on the extent of resolution of presenting clinical signs, which include the following categories: Complete: clinical signs resolved. Partial: If there was substantial but incomplete improvement of clinical signs. Failed: Dog did not improve or deteriorated further. Owner inferred outcome based on pain, mobility and quality of life scores obtained through questionnaires.
Main Findings (relevant to PICO question)	Improvement after ESI was seen in 27/32 dogs, with 14 dogs showing partial response and 13 dogs showing complete response. All five dogs which had no clinical response to ESI had subsequent decompressive surgery. Out of 14 dogs with partial response, nine relapsed, with seven having surgical decompression. Out of the 13 dogs with complete response to ESI, eight relapsed, with five having subsequent surgical decompression. Five dogs showed persistent improvement without relapse following ESI. A total of 17 dogs underwent decompressive surgery, with surgery showing a trend towards reduced pain, increased mobility, and greater quality of life score in all dogs. Complete response was seen in eight dogs and partial response in nine dogs.
Limitations:	Outcomes of dogs (complete, partial, failed) were determined by the neurologists, but information regarding the specific clinical signs displayed by each dog was not available. Clinical outcome information relied on the expertise of the same people that performed the procedures, potentiating clinician bias. Owner perceived outcome is inherently subjective, prone to caregiver placebo effect that may be impacted by the relative cost of ESI versus surgical decompression. Utilisation of a subjective numeric grading for owners perceived outcome was not based on a previously validated method.

**Ness (1994):** 

Population:	Dogs presented to the author between 1987 and 1992, which are diagnosed with degenerative lumbosacral stenosis (DLSS). Inclusion criteria: Diagnosis of DLSS based on history of low back pain for 3 weeks or longer, with or without a neurological deficit. History supported by evidence obtained from radiography and myelography. Low back pain was detected by pressing over the dorsum at lumbosacral level or by applying lordosis test. Exclusion criteria: Dogs that presented with lumbosacral disorders which was not caused by degenerative lumbosacral stenosis.
Sample size:	30 dogs.
Intervention details:	16 dogs underwent conservative treatment, with a standard protocol of exercise restriction for 8–10 weeks and administration of phenylbutazone as required at a dose not exceeding 7.5 mg/kg twice daily for up to 6 weeks. 16 dogs (inclusive of four dogs originally under conservative treatment group which failed conservative treatment) underwent surgical treatment of dorsal laminectomy. Excision of dorsal annulus and fenestration of the lumbosacral disc were performed. Unilateral facetectomy was conducted to decompress the seventh lumbar nerve in five dogs which showed marked neurological signs with lateralisation. Two dogs were withdrawn from the study as owners were not ready to begin treatment.
Study design:	Retrospective study.
Outcome studied:	Clinical outcome was assessed based on the alleviation of pain and the resolution of neurological defects. Surgical treatment outcome was considered: Good in dogs which regained preoperative activity levels (as assessed by owner). Acceptable in dogs with a persistent abnormality or requiring continued medication though otherwise active. Poor in all other cases.
Main Findings (relevant to PICO question)	Surgical treatment was successful in alleviating pain in 13/16 (81%) dogs, usually within 6 weeks of the operation. Neurological defects responded more slowly to surgery, with time taken to best recovery varying from 8–30 weeks. Out of 16 dogs treated surgically, 11 were treated by dorsal lumbosacral laminectomy and excision of the dorsal portion of the lumbosacral disc, while the other five had additional unilateral facetectomy to decompress the seventh lumbar nerve. Out of these 11 dogs who were treated by dorsal lumbosacral laminectomy and excision of the dorsal portion of the lumbosacral disc, 6/11 (54.5%) of dogs were deemed to have a good outcome, while 3/11 (27.3%) of dogs were deemed to have an acceptable outcome, 1/11 (9.1%) of dogs were deemed to have poor outcome, while 1/11 (9.1%) of dogs were lost to follow-up. Out of the five dogs who were treated by dorsal lumbosacral laminectomy and excision of the dorsal portion of the lumbosacral disc with additional unilateral facetectomy, 3/5 (60%) of dogs were deemed to have an acceptable outcome, 1/5 (20%) of dogs were deemed to have poor outcome, while 1/5 (20%) of dogs were lost to follow-up. Conservative management was considered good in 8/16 (50%) dogs.
Limitations:	There was no information provided on the initial clinical signs presented by each individual dog. No standardised method of assessing the post-surgical and post-conservative treatment outcome, leading to difficulties in comparison. No standardised review time, all dogs having different healing periods but no comparison of how the clinical status of the dogs at fixed periods after the treatment. Owner perceived outcome is inherently subjective, prone to caregiver placebo effect.

## Appraisal, application and reflection

Canine degenerative lumbosacral stenosis (DLSS) represents a syndrome in dogs that comprises of lumbosacral pain as well as neurological dysfunction in some cases. This syndrome is related to the degeneration and subsequent enlargement of the soft tissue structures of the lumbosacral junction as well as compression of the cauda equina (Worth et al., 2019). It is a common disorder which is multifactorial in origin, with intervertebral disc degeneration (IVD) being a major contributor (Meij & Bergknut, 2010). In particular, intervertebral disc protrusion caused by fibroid metaplasia as well as the development of small separations in the lamellae of the annulus fibrosus has been identified as a contributing factor to DLSS. The process of fibroid metaplasia refers to the increased collagen content and change in the notochordal cells to become more fibrocyte-like, coupled with the decreased integrity of the lamellae of the annulus fibrosus. This leads to extension of degenerative fibroid nuclear material into and between the fibres of the annulus. Eventually, the surface of the annulus fibrosus gradually thickens and protrudes, displacing the dorsal longitudinal ligament and gradually compressing the nerve roots (Fenn et al., 2020).

Possible causes of lumbosacral disease include cauda equina compression which can occur from lateral disc protrusion which compresses the sciatic nerve at the foramen level. Other causes also include synovial cysts as well as dynamic compression in lumbosacral dorsi-flexion but otherwise minimal compression in neutral position. Compression could be categorised as dynamic when instability and thus motion of the vertebral segments results in variation in severity of compression from moment to moment depending on the position of the vertebrae at a specific point in time (Jeffery et al., 2013). The spinal nerves originating from the lumbosacral section of the vertebral column stretches to the hips, stifle, tarsus, urinary bladder as well as anal and urinary sphincters, in order from L4 to S3. Neurological findings associated with degeneration at L4-S1 often presents as femoral pseudo-hyperreflexia, muscle atrophy and potentially decreased reflexes due to impingement of the femoral and sciatic nerves.

The proposed pathogenesis for canine DLSS involves disc degeneration resulting in ventral subluxation of the sacrum as well as thickening of the articular processes, which results in structural failure of the disc (Meij & Bergknut, 2010). In response to the proposed disease pathogenesis, current surgical treatments such as dorsal laminectomies are conducted to relieve pressure on the cauda equina and foraminotomies are performed with the aim of decompressing the sciatic nerve and releasing nerve roots which are entrapped. The type of surgery indicated would depend on the direction of protrusion, with dorsal laminectomies being indicated for dorsal ventral disc protrusion and foraminotomies being indicated for foraminal protrusions. Presence of ventral subluxation of S1, which refers to the S1 disc being more ventrally displaced as compared to neighbouring vertebrae, as well as dynamic compression, often indicates the need for fixation and fusion stabilisation to reduce the abnormal mobility of the vertebrae which was identified as instability.

It has been speculated that cell-mediated inflammatory responses occur upon disc damage, which results in ingrowth of blood vessels and nerves into the damaged disc, exacerbating lumbosacral pain. Nonsurgical treatments for DLSS work to mediate the inflammatory responses that occur during disc damage, often using nonsteroidal anti-inflammatory drugs (NSAIDs), a change in exercise pattern as well as reduction in body weight. In more recent times, lumbosacral epidural injections of corticosteroids have been reported to be effective in improving symptoms for dogs with DLSS.

Only two studies out of the many focusing on treatment of DLSS compared the efficacy of nonsurgical treatment compared to surgical treatment. In the first study, Gomes et al. (2020) evaluated the efficacy of epidural steroid injection (ESI) of methylprednisolone acetate. Out of 32 dogs that received the ESI, only five experienced persistent improvement without relapse, while 17 dogs ultimately underwent decompressive surgery. In this study, epidural steroid injection appears to only be successful in the long-term in a small number of dogs. In the second study, Ness (1994) evaluated the clinical outcomes of 16 dogs being treated surgically against 16 dogs treated under conservative treatment. In this study, surgical treatment was successful in alleviating pain in 13/16 81.3% of dogs, while conservative treatment was considered good in 8/16 (50%) of dogs. While this study attempted to compare clinical outcomes of surgically and conservatively treated dogs with DLSS, the outcome measures are not standardised, and it was not mentioned clearly what ‘good’ referred to in the dogs being conservatively treated. It is to be noted that the author conducted a facetectomy in five surgically treated cases, a technique which carries risks of exacerbating existing instabilities (Bebchuk, 2017).

With regards to papers published focusing on the clinical outcomes of dogs treated using surgical treatment only, Hankin et al. (2012) evaluated dogs with DLSS that had undergone transarticular facet screw stabilisation and dorsal laminectomy for long-term outcome through a retrospective study. Follow-up radiographs of the surgical site showed evidence of bone healing and stabilisation of the distracted lumbosacral intervertebral disc (IVD) space in all 15 dogs available for long-term follow-up. 11/13 (84.6%) owner questionnaires returned also demonstrated that the dog had regained normal ability to run and jump without perceptible lameness, signaling that the surgical procedure was highly successful. Gomes et al. (2018) evaluated the long-term outcome of dogs with DLSS that have undergone lateral foraminotomy through a retrospective study and 33/34 (97.1%) of the cases showed a long-term complete resolution of clinical signs. Golini et al. (2012) performed a retrospective study on dogs with DLSS which had undergone dorsal laminectomy and transarticular fixation of the facet joints using screws instead of pins. Clinical outcome improved in 13/17 (76.5%) dogs. As compared to a study conducted by Hankin et al. (2012) which involved the same surgical procedure with a success rate of 23/26 (88.5%) in terms of improvement in clinical outcome, success rate appears to be lower at 13/17 (76.5%). Despite the slight differences in success rates, transarticular facet screw stabilisation and dorsal laminectomy appears to be mostly effective in improving clinical outcome long-term in dogs with DLSS. Danielsson & Sjöström (1999) monitored the long-term outcome of dogs with DLSS that had undergone dorsal laminectomy and dorsal fenestration, with 122/131 (93.1%) of the dogs showing improvement clinically within the follow-up period.

With regards to papers published focusing on the clinical outcomes of dogs treated using nonsurgical treatment only, De Decker et al. (2014) evaluated the difference in clinical outcome for dogs treated nonsurgically through restricted exercise in combination with anti-inflammatory and analgesic drugs for DLSS. It was found that 17/21 dogs on medical management had obvious improvement or resolution of clinical signs, which makes up 81%. Based on this study, it appears that conservative treatment has reasonable success in resolving lower back pain and neurological deficits in the long-term. In a case report by Mrkovaĉki et al. (2021), a dog diagnosed with DLSS was injected with cultured autologous adipose tissue-derived mesenchymal stem cells (AT-MSCs) bilaterally at the level of L7-S1. Clinical outcome 60 days post AT-MSC treatment was positive, with absence of pain upon L7-S1 region palpation and negative lordosis test. Movement was also normal without stiffness of the hindlimbs, with preservation of all neurologic reflexes. However, due to the small sample size (n = 1), there is a need for further studies to be conducted on this treatment method to gather more meaningful data for widespread clinical use.

In conclusion, while it appears that surgical treatment does resolve lumbosacral pain and neurological dysfunction in the long-term in a proportion of dogs with DLSS, it is important to note that there is a serious lack of studies conducted on dogs diagnosed with DLSS who have undergone nonsurgical treatment, although this is a very common form of treatment in dogs diagnosed with DLSS today. It is generally understood that dogs with severe DLSS tend to have higher rates of improvement with surgical treatment, though a standardised grading system for DLSS according to clinical signs as well as pathogenesis would be helpful in streamlining dogs for further studies. Investigations into how the underlying pathogenesis of DLSS correlates with the success rate in surgical and / or nonsurgical treatment would greatly benefit from a DLSS grading system. For surgical treatments, it is pertinent for the specific part of lumbosacral failure to be identified in dogs admitted to the sample population so that future evaluation of the efficacy of surgical treatments can be more targeted and allow for more informed decision-making in clinical practice. There should also be the establishment of a standardised pet owner questionnaire to be used across all DLSS studies regarding the post-treatment condition of their dog. With these tools made available, more randomised control studies then need to be carried out.

While considering the best type of treatment for long-term resolution of DLSS, it is crucial to appreciate that lumbosacral disease waxes and wanes. In the case of severe uncontrollable pain, surgery is indicated with the purpose of controlling the pain using the shortest time possible, though it does sometimes resolve the signs of lumbosacral disease in the long-term. Due to the current lack of reliable and strong evidence for the long-term outcome for dogs with DLSS that had undergone surgical and nonsurgical treatment of DLSS respectively, it cannot be confidently ascertained that surgical treatment is more effective than nonsurgical treatment in reducing lower back pain and neurological dysfunction in the long-term.

## Methodology

**Search strategy:** 

Databases searched and dates covered:	CAB Abstracts via Web of Science Platform 1973–Week 11 2023 Medline via Ovid 1946–Mar 16 2023
Search strategy:	CAB Abstracts: (canine OR dog OR dogs OR canines) AND (DLSS OR Degenerative Lumbosacral Stenosis OR Degenerative Lumbosacral Disease) AND (Surgical Treatment OR Surgical Decompression OR Dorsal Laminectomy OR Foraminotomy) AND (Non-Surgical Treatment OR Medical Treatment OR Conservative Treatment OR Epidural Steroid Injection) AND (Lumbosacral Pain OR Neurological Dysfunction OR Neurological signs) Medline via Ovid: (canine OR dog OR dogs OR canines) AND (Degenerative Lumbosacral Stenosis OR DLSS OR Degenerative lumbosacral disease) AND (Treatment OR Management)
Dates searches performed:	16 Mar 2023

**Exclusion / inclusion criteria:** 

Exclusion:	Papers inappropriate to the PICO (non-related title and abstract; other species; papers unable to demonstrate long-term post-treatment outcome), papers not accessible.
Inclusion:	Papers identifying the long-term outcomes following surgical and medical / nonsurgical treatment of canine degenerative lumbosacral disease.

**Search outcome:** 

Database	Number of results	Excluded – Not in English language	Excluded – Non-relevant to PICO	Excluded – Unable to access	Total relevant papers
CAB Abstracts					
PubMed					
Total relevant papers when duplicates removed					

## ORCiD

Kristy Goh Rui Qi: https://orcid.org/0000-0003-3938-8543

## Conflict of interest

The author declares no conflicts of interest.

## References

[BIBR-1] Bebchuk T. (2017). Lumbosacral Decompression and Foraminotomy. Current Techniques in Canine and Feline Neurosurgery.

[BIBR-2] Danielsson F., Sjöström L. (1999). Surgical Treatment of Degenerative Lumbosacral Stenosis in Dogs. Veterinary Surgery.

[BIBR-3] Decker S., Wawrzenski L., Volk H. (2014). Clinical signs and outcome of dogs treated medically for degenerative lumbosacral stenosis: 98 cases (2004–2012. Journal of the American Veterinary Medical Association.

[BIBR-4] Fenn J., Olby N. (2020). & the Canine Spinal Cord Injury Consortium (CANSORT-SCI. Frontiers in Veterinary Science.

[BIBR-5] Golini L., Kircher P.R., Lewis F.I., Steffen F. (2012). Transarticular Fixation With Cortical Screws Combined With Dorsal Laminectomy and Partial Discectomy as Surgical Treatment of Degenerative Lumbosacral Stenosis in 17 Dogs: Clinical and Computed Tomography Follow-Up. Veterinary Surgery.

[BIBR-6] Gomes S.A., Lowrie M., Targett M. (2018). Long-term outcome following lateral foraminotomy as treatment for canine degenerative lumbosacral stenosis. Veterinary Record.

[BIBR-7] Gomes S.A., Lowrie M., Targett M. (2020). Single dose epidural methylprednisolone as a treatment and predictor of outcome following subsequent decompressive surgery in degenerative lumbosacral stenosis with foraminal stenosis. The Veterinary Journal.

[BIBR-8] Hankin E., Jerram R., Walker A., King M., Warman C. (2012). Transarticular Facet Screw Stabilization and Dorsal Laminectomy in 26 Dogs with Degenerative Lumbosacral Stenosis with Instability. Veterinary Surgery.

[BIBR-9] Jeffery N., Levine J., Olby N., Stein V. (2013). Intervertebral disk degeneration in dogs: consequences, diagnosis, treatment, and future directions. Journal of Veterinary Internal Medicine.

[BIBR-10] Meij B.P., Bergknut N. (2010). Degenerative lumbosacral stenosis in dogs. The Veterinary Clinics of North America: Small Animal Practice.

[BIBR-11] Mrkovaĉki J., Srzentić Dražilov S., Spasovski V., Fazlagić A., Pavlović S., Nikĉević G. (2021). Case Report: Successful Therapy of Spontaneously Occurring Canine Degenerative Lumbosacral Stenosis Using Autologous Adipose Tissue-Derived Mesenchymal Stem Cells. Frontiers in Veterinary Science.

[BIBR-12] Ness M.G. (1994). Degenerative lumbosacral stenosis in the dog: A review of 30 cases. Journal of Small Animal Practice.

[BIBR-13] Worth A., Meij B., Jeffery N. (2019). Canine Degenerative Lumbosacral Stenosis: Prevalence, Impact And Management Strategies. Veterinary Medicine (Auckland.

